# Outcomes for women with diabetes admitted for labour care to midwifery units in the UK: a national prospective cohort study and survey of practice using the UK Midwifery Study System (UKMidSS)

**DOI:** 10.1136/bmjopen-2024-087161

**Published:** 2024-12-03

**Authors:** Alessandra Morelli, Lisa Smith, Amar Karia, Amber Marshall, Rachel Plachcinski, Wendy Tyler, Rachel Rowe

**Affiliations:** 1 NIHR Policy Research Unit in Maternal and Neonatal Health and Care, National Perinatal Epidemiology Unit, Nuffield Department of Population Health, University of Oxford, Oxford, UK; 2 London Ambulance Service NHS Trust, London, UK; 3 Royal College of Obstetricians and Gynaecologists, London, UK; 4 Founder, bigbirthas.co.uk, Stonehouse, UK; 5 Independent Parent, Patient and Public Involvement and Engagement Consultant, Dewsbury, UK; 6 Consultant Neonatologist (Retired), Shrewsbury, UK

**Keywords:** Midwifery, Diabetes in pregnancy, Pregnant Women, Pregnancy, OBSTETRICS

## Abstract

**Abstract:**

**Objectives:**

To describe outcomes in women admitted for labour care to midwifery units with gestational or pre-existing diabetes, compare outcomes with other women admitted to the same units and describe admission and care guidance in midwifery units typically admitting women with diabetes.

**Design:**

A national cohort study and a survey of practice.

**Setting:**

We used the UK Midwifery Study System to collect data from midwifery units in the UK between October 2021 and February 2023.

**Participants:**

Women with a diagnosis of diabetes admitted for labour care to a midwifery unit were compared with a cohort of women without diabetes admitted for labour care to the same units.

**Primary and secondary outcome measures:**

The primary outcome was a composite measure of maternal outcome reflecting the need for obstetric care (one or more of augmentation, instrumental birth, caesarean birth, maternal blood transfusion, third or fourth-degree perineal tear, maternal admission to higher level care). We also investigated a number of secondary maternal and neonatal outcomes.

**Results:**

Overall, 420 (0.7% (95% CI 0.67% to 0.82%) of the 56 648 women admitted to midwifery units in the study period were recorded as having diabetes, most (84%) with diet-controlled gestational diabetes. Women with diabetes were no more likely than comparison women to experience the composite primary outcome (18.7% vs 20.7%, adjusted relative risk=1.31, 95% CI 0.96 to 1.80). We found no statistically significant differences between the two groups for the secondary maternal and neonatal outcomes investigated: augmentation, postpartum haemorrhage >1.5 L, shoulder dystocia, maternal blood transfusion and maternal admission for higher level care, Apgar <7 at 5 min, initiation of breast feeding and neonatal unit admission.

**Conclusions:**

The findings of this study provide evidence that selected women with well-controlled gestational diabetes may safely plan birth in midwifery units on the same site as obstetric and neonatal services. With clear admission criteria and careful care planning, access to a midwifery unit provides an opportunity to increase choice, reduce intervention and improve outcomes for these women.

**STRENGTHS AND LIMITATIONS OF THIS STUDY**

- The national population-based design of this study reduces the risk of the biases associated with local, hospital-based studies.
- Most midwifery units (MUs) in the UK participated in the study, reducing the possibility of selection bias.
- Because over 99% of the diabetes group had gestational diabetes (with only eight women on insulin) and most (93%) of the diabetes group were admitted to alongside MUs (AMUs), our results should be considered as most generalisable to women with diet-controlled gestational diabetes mellitus or those on metformin, considering birth in an AMU.
- Our study was underpowered for the primary outcome as the sample size was lower than expected.

## Background

 Diabetes is the most common endocrine disorder in the general population and its incidence is rising across the world.^1^ It is characterised by reduced production of insulin by the pancreas or decreased sensitivity of receptors to insulin, resulting in elevated levels of glucose in the blood as a consequence of abnormal carbohydrate metabolism.^1^ Gestational diabetes mellitus (GDM) is defined as ‘any degree of glucose intolerance with onset or first recognition during pregnancy’.^2^ Around 2%–5% of pregnant women in England and Wales are affected by pre-existing diabetes or GDM and this figure is increasing.^3^

Diabetes in pregnancy is associated with a range of complications and adverse birth outcomes including macrosomia, large for gestational age, pre-eclampsia, shoulder dystocia and perinatal mortality, and increased rates of induction and caesarean birth.^3 4^ At the time of this study, national guidelines advised women with diabetes (pre-existing or GDM), to plan birth in an obstetric unit (OU), where ‘advanced neonatal resuscitation skills are available 24 hours a day’ .^3 5^ A recent change to national guidelines means that birth in an OU is now advised for women being treated with medication for diabetes (pre-existing or GDM).^6^ In women who are healthy with straightforward pregnancies, planning birth in a midwifery unit (MU) is considered safe and is associated with a more positive experience and a reduced risk of medical intervention, compared with planned OU birth.^6^ MUs are healthcare facilities where midwives lead on the provision of care for pregnant women and during childbirth. In the UK, there are two types of MU: ‘alongside’ (AMU), located on the same site as an OU or ‘freestanding’ (FMU) facilities on a geographically separate site from an OU.^7^ A 2019 survey of UK MUs found that almost 25% of local National Health Service (NHS) admission guidelines for MUs indicated that women with GDM were explicitly eligible for planned birth in an MU (3%) or would be considered for admission (21%), while others reported admission criteria in relation to diabetes that were in line with National Institute for Health and Care Excellence (NICE) guidance at the time.^8^ This variation in practice in part reflects insufficient evidence about the outcomes for women with diabetes planning birth in MUs.

A Diabetes and Pregnancy Research Priority Setting Partnership, conducted in 2019 with women and healthcare professionals, identified that the labour and birth experiences of women with diabetes and how to enhance choice for these women was a ‘top-ten’ priority.^9^ The ‘long-list’ of questions contributing to this priority topic included ‘When is it safe for women with diabetes to give birth in a MU compared with a hospital birth?’. There is no evidence about how many women with diabetes (pre-existing or GDM) are admitted to MUs for labour care, about their characteristics and outcomes for women and their babies. Consequently, it is unclear whether some women with diabetes might safely plan birth in an MU, particularly an AMU where neonatal and obstetric services are readily accessible.

In this study, we aimed to provide evidence to inform birthplace decision-making for women with diabetes in pregnancy. We addressed this with three specific objectives: (a) to explore and describe clinical characteristics, labour care, and maternal and perinatal outcomes, in women with diabetes admitted for labour care to an MU in the UK; (b) to compare outcomes in this group with women without a diagnosis of diabetes in their current pregnancy and admitted for labour care to the same MUs and (c) to describe guidance about care of women with diabetes and their babies in MUs typically admitting women with diabetes.

## Methods

We carried out a national population-based cohort study (the ‘Diabetes Study’), identifying and collecting data about all women with a diagnosis of diabetes (pre-existing or GDM) in their current pregnancy, admitted for labour care in all MUs across the UK NHS between 1 October 2021 and 30 September 2022, and a comparison cohort of women who had not had a diagnosis of diabetes in their current pregnancy, matched on time of admission to the same MUs.

We also carried out a post hoc survey of practice (the ‘Diabetes Survey’) between 29 September 2022 and 1 February 2023. This was sent to selected MUs with relatively higher numbers of women admitted with diabetes, as indicated by data collected in the Diabetes Study.

For both, data were collected using the UK Midwifery Study System (UKMidSS), a research infrastructure covering 199 MUs, 126 AMUs and 73 FMUs, in the UK in 2021–22. UKMidSS methods have been described elsewhere.^10^

### Diabetes study

#### Data collection

In each MU, one or more midwife ‘reporters’ received monthly emails from the UKMidSS co-ordinating centre at the National Perinatal Epidemiology Unit in the University of Oxford. In response, they reported the number of women with a diagnosis of diabetes who were admitted for labour care to the MU (including zero if they had no women with diabetes to report). They also reported data about total admissions and births in the MU each month and whether the unit had been closed to admissions for the whole month.

On reporting a woman who had diabetes, electronic case report forms (ECRFs) were automatically generated. All data were entered directly from women’s notes and/or hospital electronic patient records into the ECRF. Data collected on the ECRF included confirmation of eligibility, previous and current obstetric history, sociodemographic and clinical characteristics, pregnancy and labour care, and maternal and neonatal outcomes. Reporters also identified and entered data about a comparison cohort, selected as the woman without a current diagnosis of diabetes, admitted to the MU immediately before each woman who had diabetes.

Email reminders were sent for overdue monthly reports and outstanding data entry. Further monthly status report emails summarised reporting and data entry completion and listed data queries about missing or invalid data which were generated automatically in the ECRF.

#### Outcomes

The primary outcome was a composite measure of maternal outcome, which reflected the need for obstetric care, comprising at least one of the following: augmentation with oxytocin, instrumental birth, intrapartum caesarean birth, general anaesthesia, maternal blood transfusion, third/fourth-degree perineal tear and maternal admission for higher level care (high dependency/enhanced/intensive care) in the immediate postnatal period.^11^

We also investigated a number of secondary outcomes. The maternal outcomes investigated were the individual components of the composite outcome; transfer from the MU to the care of an obstetrician during labour or within 24 hours of birth; documented shoulder dystocia; immersion in water during labour; birth in water; ‘straightforward vaginal birth’ (ie, birth without forceps, ventouse or caesarean, with no third/fourth-degree perineal tear and no blood transfusion)^12^; caesarean birth; postpartum haemorrhage ≥1500 mL; maternal death. While most of these are described as ‘maternal’ outcomes, some also have potential implications for neonatal well-being, for example, instrumental birth, caesarean birth, shoulder dystocia.

The neonatal outcomes investigated were Apgar score <7 at 5 min; initiation of breast feeding; neonatal unit admission; stillbirth/neonatal death.

#### Data and definitions

The diabetes group comprised women admitted for labour care in an MU who were recorded in their notes as having a diagnosis of diabetes, either GDM or pre-existing, in the current pregnancy. Women fitting these criteria, and who went on to give birth in the same admission were included, irrespective of where they gave birth.

We collected the following data about the group of women with diabetes: diabetes type (GDM or pre-existing type 1/2); whether they had an oral glucose tolerance test (OGTT), or haemoglobin A1c (HbA1C) test performed in pregnancy; the number of these tests and their results. We also collected data about whether the woman had glycaemic testing during labour and whether the baby was monitored for hypoglycaemia following birth.

For all women, we collected data about any complications in a previous pregnancy (GDM, pre-existing diabetes, baby’s birth weight >4.5 kg, shoulder dystocia, other); medical conditions known prior to the start of labour care (essential hypertension, cardiac disease, thromboembolic disorder, atypical antibodies, hyperthyroidism, renal disease, epilepsy, other); current pregnancy risk factors (in addition to diabetes for the diabetes group) identified prior to admission (body mass index (BMI) >35 kg/m^2^, anhydramnios, polyhydramnios, suspected fetal growth restriction, suspected macrosomia, group B Streptococcus, post-term pregnancy, anaemia, antepartum haemorrhage, pre-eclampsia/pregnancy-induced hypertension, malpresentation, other) and ‘complicating conditions’ identified at the start of care in labour (eg, prolonged rupture of membranes), using the list of complications indicating need for transfer to obstetric-led care in national guidelines.^5^

We derived the three-class version of the National Statistics Socio-economic Classification, using the ‘simplified method’,^13^ from the woman’s occupation (or her partner’s where the woman was out of work or where her occupation was not known), including categories for ‘employed, but occupation unrecorded’ and ‘employment status not recorded’. To derive a measure of area deprivation, UKMidSS reporters entered women’s postcodes into a bespoke ‘look-up’ website which returned a ‘score’ for the Children in Low-income Families Local Measure, which they then entered into the ECRF with other data. This score represents the proportion of children living in families in receipt of out-of-work benefits or tax credits where their reported income was less than 60% of UK median income, based on the local area in which they live.^14^ Cut-offs derived using data on the number of babies in 2018 in the UK from official birth records were used to create deciles and quintiles.

#### Statistical analysis

We estimated the prevalence of diabetes among women admitted for labour care in MUs using the total reported number of women admitted for labour care to MUs as the denominator and the total number of confirmed women with diabetes as the numerator, with 95% CIs.

We described the maternal sociodemographic and clinical characteristics, and maternal and neonatal outcomes of the diabetes group and the comparison cohort using frequencies and percentages. For the diabetes group, we also described antenatal testing for diabetes, medication use in pregnancy, glycaemic monitoring in labour and neonatal hypoglycaemia monitoring and feeding method, in the same way.

We used log Poisson regression to calculate the relative risk (RR) of the primary and secondary outcomes in the diabetes cohort relative to the comparison group adjusted RR (aRR) for maternal age, ethnic group, area deprivation quintile (Children in Low-Income Families Local Measure), gestation at admission, the presence of pre-existing medical ‘risk factors’ and previous pregnancy complications, and parity where possible (see online supplemental table S1 for categorisation). We did not adjust for current pregnancy complications as we considered that many of these could conceivably be on the ‘causal pathway’ between diabetes and the outcome.

For all analyses using the primary outcome, we used p<0.05 to assess statistical significance and, because of multiple testing, for all secondary outcomes we used p<0.01; absolute p values are reported throughout. We used Stata V.15 SE^15^ for all analyses.

#### Sample size and power

Analysis of the comparison group in the UKMidSS Severe Obesity Study gave an estimated prevalence of GDM in women admitted for labour care in AMUs of approximately 0.5% and a lower prevalence of pre-existing diabetes.^11^ Allowing for a small increase in the prevalence of GDM in women admitted for labour care in MUs as a result of changing admission criteria,^8^ we estimated that the overall prevalence of any diabetes would be around 0.8%. Using ‘denominator data’ on the number of women admitted to AMUs and FMUs from previous studies and collecting data for 12 months, we estimated that we would identify approximately 640 women in the ‘diabetes’ cohort and the same number in the comparison cohort. For the primary outcome, with an estimated incidence of 20% in the comparison group, this would give 80% power at the 5% level of significance to detect an RR of 1.3 or greater in the diabetes group.

The actual number of women in the diabetes and comparison groups generated 80% power at the 5% level to detect an RR of 1.4 or greater for the primary outcome in the diabetes group, and 2.0 or greater for a less common outcome, with an incidence of 5% in the comparison group.

### Diabetes survey

#### Data collection

For the Diabetes Survey, UKMidSS reporters in participating MUs with more than five cases or where cases were greater than 2% of admissions (whichever was greater), were invited to take part in a short survey sent out by email on 29 September 2022. The invitation email contained a unique access hyperlink to the survey hosted by the online platform LimeSurvey.^16^ The survey asked UKMidSS reporters to describe their local MU/NHS organisation guidance about (a) admission of women with diabetes to the MU; (b) intrapartum and postnatal care of women with diabetes and their babies in the MU and (c) any local initiatives to promote access for women with diabetes to midwifery-led care. Reporters were also invited to upload any relevant local guidelines. Reminders to complete the survey were sent to non-responders weekly until the survey was closed in February 2023.

#### Statistical analysis

Descriptive analysis, reporting frequencies and percentages, was used to summarise responses to the survey.

## Results

### Diabetes study

#### Response and prevalence of diabetes

A total of 186 (116 AMUs and 70 FMUs) MUs in the UK participated in the study (93% of eligible units), with 90% response to monthly report requests.

**Figure 1:**
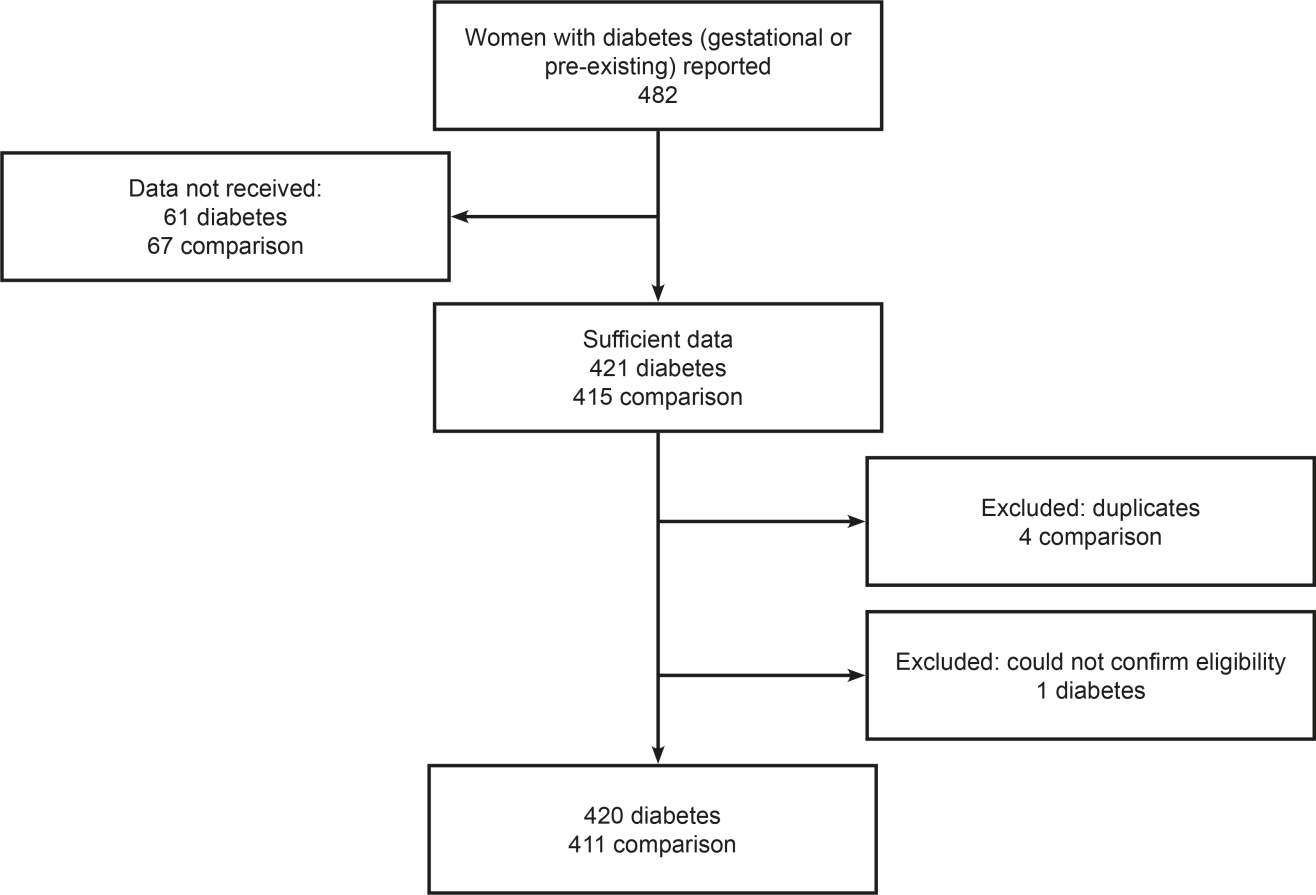
Flow diagram illustrating reported and confirmed cases of women with diabetes (either gestational or pre-existing) and comparison cohort.

Over the 12-month study period, 482 women with a diagnosis of diabetes in the current pregnancy were reported (figure 1). After exclusions, there were 420 women with diabetes for whom sufficient data were received. A total of 56 648 women were admitted to an MU over the same 12-month period, meaning that overall 0.7% (95% CI 0.67% to 0.82%) of all women admitted to MUs were recorded as having diabetes in the current pregnancy. In total, 66 MUs (35%) reported at least one confirmed woman admitted with a diagnosis of diabetes during the study period, but these ‘cases’ of diabetes were not uniformly distributed across MUs. Among the 116 AMUs, 57 (49%) reported at least one woman with a diagnosis of diabetes, giving a ‘prevalence’ in AMUs of 0.7% (95% CI 0.67% to 0.82%). However, 74% of all women reported with a diagnosis of diabetes in AMUs were ‘clustered’ in 12 units, around half of which were in one NHS region. Among the 70 FMUs, there were 9 (13%) reported women with a diagnosis of diabetes, for a prevalence in FMUs of 0.7% (95% CI 0.48% to 1.03%). In total, 392 (93%) women with diabetes were admitted to an AMU and 28 (7%) to an FMU during the study period.

### Maternal characteristics

#### Sociodemographic and clinical characteristics

Compared with the comparison group, women with diabetes were more likely to have a BMI ≥30 kg/m^2^, be aged over 30 years, be of Asian ethnicity, have given birth before, have lower gestation at birth and to give birth to a baby weighing less than 3500 g (table 1).

**Table 1:** Sociodemographic and clinical characteristics in diabetic and comparison women

	Diabetes n=420		Comparison n=411		All n=831	
	n	%	n	%	n	%
BMI at booking (kg/m^2^)						
<18.5	13	3.1	13	3.2	26	3.2
18.5–24.9	178	42.9	222	55.1	400	48.9
25–29.9	128	30.8	124	30.8	252	30.8
30–35.0	70	16.9	38	9.4	108	16.9
>35	26	6.3	6	1.5	32	3.9
Missing	5		8		13	
Maternal age (years)						
Under 20	0	0	12	2.9	12	1.4
20–24	29	6.9	51	12.4	80	9.6
25–29	84	20.0	108	26.3	192	23.1
30–34	177	42.1	150	36.5	327	39.3
35–39	114	27.1	75	18.3	189	22.7
40+	16	3.8	15	3.6	31	3.7
Missing	0		0	0	0	
Ethnic group						
White	213	50.9	253	62.0	466	56.4
Asian	149	33.6	91	22.3	240	29.0
Black	32	7.6	41	10.1	73	8.8
Other	25	6.0	23	5.6	48	5.8
Missing	1		3		4	
Socioeconomic status						
Higher managerial, admin, prof	141	35.5	146	37.8	287	36.7
Intermediate	116	29.2	84	21.8	200	25.5
Routine and manual	79	19.9	89	23.1	168	21.5
Unemployed/student	20	5.0	34	8.8	54	6.9
Employed but unrecorded	41	10.3	33	8.6	74	9.5
Missing	23		25		48	
Area deprivation quintile						
1st	64	15.6	79	19.7	143	17.6
2nd	81	19.8	70	17.4	151	18.6
3rd	92	22.4	86	21.4	178	21.9
4th	111	27.1	105	26.1	216	26.6
5th	62	15.1	62	15.4	124	15.3
Missing	10		9		19	
Smoking status						
Non-smoker during pregnancy	393	93.6	382	92.9	775	93.3
Smoker during pregnancy	19	4.5	22	5.4	41	4.9
Not recorded	8	1.9	7	1.7	15	1.8
Missing	0		0		0	
Parity*						
0	151	36.0	187	45.5	338	40.7
1	172	41.0	146	35.5	318	38.3
2	68	16.2	49	11.9	117	14.1
3 or more	29	6.9	29	7.1	58	6.7
Missing	0		0		0	
Gestation at admission (weeks)						
36–37	26	6.7	13	3.5	39	5.2
38	90	23.3	46	12.5	136	18.1
39	147	38.1	111	30.3	258	34.3
40	106	27.5	114	31.1	220	29.2
41–42	17	4.4	83	22.6	100	13.3
Missing	34		44		78	
Birth weight (g)						
<3000	92	21.9	68	16.6	160	19.3
3000–3499	198	47.1	174	42.3	372	44.8
3500–3999	104	24.8	131	31.9	235	28.3
≥4000	26	6.2	38	9.3	64	7.7
Missing	0		0		0	

* Number of previous pregnancies carried to at least 24 completed weeks’ gestation.

BMIbody mass index

In terms of clinical risk characteristics, all but three of the women in the diabetes group had GDM. Of the three (0.7%) women with pre-existing diabetes, one had type 2, and two did not have the type recorded. Women with a diagnosis of diabetes were more likely to have had one or more previous pregnancy complication, of which the most commonly occurring was previous GDM, and to have one or more current pregnancy risk factor in addition to diabetes (table 2).

**Table 2:** Clinical ‘risk’ characteristics

	Diabetes n=420		Comparison n=411		All n=831	
	n	%	n	%	n	%
Medical risk factors*						
None	387	92.4	387	94.4	774	93.4
One or more	32	7.6	23	5.6	55	6.6
Previous pregnancy complications†						
None	159	59.1	181	81.2	340	69.1
One or more	110	40.9	42	18.9	152	30.9
Current pregnancy risk factors‡						
None	346	82.6	359	87.6	705	85.0
One or more	73	17.4	51	12.4	124	15.0
‘Complicating conditions’ identified at start of labour care						
None	387	92.6	389	94.9	776	93.7
One or more	31	7.4	21	5.1	52	6.3
Selected specific risk factors						
Previous GDM	77	18.3	6	1.5	83	10.0
Pre-existing diabetes§	3	0.07	0	0.0	3	0.04
Diabetes type						
GDM	417	99.3				
Pre-existing type 2	1	0.2				
Not recorded	2	0.5				

* Hypertension, confirmed cardiac disease, thromboembolic disorder, atypical antibodies, hyperthyroidism, diabetes, renal disease, epilepsy.

† GDM, Ppre-existing diabetes, Mmacrosomic baby >4.5 kg, Sshoulder dystocia.

‡ In addition to diabetes., BMI at booking >35, Ppost-term (>42 weeks) Aanaemia, Ggroup B Streptococcus, Aantepartum haemorrhage, Ppre-eclampsia/pregnancy-induced hypertension, GDM, Mmalpresentation (breech or transverse lie), multiple pregnancy.

§ One woman with pre-existing type 2 and two women with diabetes of an unrecorded type were included women with diabetes type not recorded.

BMIbody mass indexGDMgestational diabetes mellitus

### Diabetes screening, medication use and monitoring in labour

#### Diabetes screening and medication in pregnancy

Most women in the group with diabetes had an OGTT performed in pregnancy (315, 76%), with 38 women (13%) having more than one. Around 50% of women had an HbA1C test during pregnancy, with only four (2%) having a level greater than 47 mg/dL. Of the women who had an HbA1C test in pregnancy, 58 (14%) women had only an Hba1C and no OGTT (online supplemental table S2). Of those, 16 (28%) had previous GDM and 42 (72%) did not.

In the diabetes group, 65 (16%) women received medication for diabetes, with most of them (57, 88%) receiving metformin, and a small number insulin (2, 3%) or both (6, 9%) (online supplemental table S3).

#### Diabetes monitoring in labour

In the diabetic group, 249 (59%) women had a documented plan for monitoring in labour. Glycaemic monitoring was performed in 226 (46%) women with diabetes. For women in whom glycaemic monitoring in labour did not take place this was most often because their labour was short with insufficient time for monitoring (69%). Where glycaemic monitoring took place, this was mostly carried out by staff (143, 63%), with around a quarter (26%), having a mixture of staff and self-monitoring, and a small proportion of women self-monitoring only (11%) (online supplemental table S4).

### Maternal outcomes

#### Primary outcome

Among the diabetic group, 87 women (21%) experienced our composite primary outcome, comprising at least one of augmentation, instrumental birth, caesarean birth, maternal blood transfusion, third or fourth-degree perineal tear and maternal admission to higher level care, compared with 77 (19%) in the comparison group (aRR 1.31, 95% CI 0.96 to 1.80) (table 3).

**Table 3:** Primary outcome

	Events	Births			Unadjusted		Adjusted*	
	n	n	%	95% CI	RR	95% CI	RR	95% CI
Composite maternal outcome†								
Comparison group	77	411	18.7	15.1 to 22.8			1	
Diabetes group	87	420	20.7	16.9 to 24.9	1.1	0.86 to 1.41	1.31	0.96 to 1.80

* Adjusted for maternal age, ethnic group, Cchildren in Llow I-income Ffamilies Mmeasure quintile, gestation at admission, previous pregnancy complications, medical risk factors, and parity.

† Comprising: augmentation, instrumental birth, caesarean, maternal blood transfusion, 3rd/4th degree tear, maternal admission to higher level care.

#### Secondary maternal outcomes

After adjustment for other factors, the diabetes group had a higher risk of experiencing any transfer during labour or after birth (25% vs 23%; aRR 1.27, 99% CI 0.88 to 1.82) (see online supplemental table S5 for reasons for transfer); a caesarean birth (6% vs 5%; aRR 1.74, 99% CI 0.55 to 5.52); a third or fourth-degree tear (4% vs 3%; aRR 1.54, 99% CI 0.63 to 3.73); shoulder dystocia (1% vs 1%; aRR 1.67, 99% CI 0.95 to 29.5) and admission for higher level care (5% vs 4%; aRR 1.23, 99% CI 0.04 to 3.56), but absolute differences were typically small and none were statistically significant (online supplemental table S6). There were no maternal deaths recorded in our cohort.

### Neonatal outcomes

The number and proportion of babies with a 5 min Apgar score <7 were the same in both groups (table 4). The proportion of women who initiated breast feeding was also similar in the diabetes and comparison groups. Babies born to women in the diabetes group had a higher risk of being admitted to a neonatal unit (3% vs 2%; aRR 1.64, 95% CI 0.55 to 4.84), but this was not statistically significant.

**Table 4:** Secondary neonatal outcomes

	Events	Births			Unadjusted		Adjusted*	
	n	n	%	95% CI	RR	99% CI	RR	99% CI
Apgar score <7 at 5 min								
Comparison group	3	407	0.7	0.2 to 2.1	1		1	
Diabetes group	3	414	0.7	0.1 to 2.1	0.98	0.08 to 12.70	0.82	0.06 to 10.84
Neonatal unit admission								
Comparison group	10†	410	2.4	1.2 to 4.4	1		1	
Diabetes group	14‡	419	3.3	1.8 to 5.5	1.37	0.48 to 3.93	1.64	0.55 to 4.84
Breast feeding								
Comparison group	362	411	88.1	85.0 to 91.0	1		1	
Diabetes	375	420	89.3	81.9 to 94.8	1.01	0.96 to 1.07	1.02	0.98 to 1.07

* Adjusted for: parity, maternal age, ethnicity, socioeconomic status, area deprivation quintile, smoking status, birthweightbirth weight, gestation at admission, BMI, previous pregnancy complications, medical risk factors where appropriate. For some outcomes, not adjusted for all potential confounders because of small numbers.

† Reason for neonatal unit admission: respiratory problems (3), suspected perinatal asphyxia (2), suspected infection (3), meconium aspiration (1), jaundice (1).

‡ Reason for neonatal unit admission: respiratory problems (4), suspected perinatal asphyxia (2), hypoglycaemia (2), Ssuspected infection (3), meconium aspiration (1), bilious vomiting (2).

BMIbody mass index

There were no stillbirths or neonatal deaths in the cohort.

### Neonatal hypoglycaemia monitoring and feeding method

In the diabetes cohort, 392 babies (94%) were monitored for neonatal hypoglycaemia. Of those 320 (82%) received breastmilk at their first feed, and 279 (71%) were exclusively breastfed during the hypoglycaemic protocol. In the comparison group, 15 babies (4%) were monitored for hypoglycaemia and of those 11 (73%) were exclusively breastfed (online supplemental table S7).

### Diabetes survey results

A total of 10 units from 8 NHS organisations were eligible to receive the Diabetes Survey, and 8 (80%) units (all AMUs) from the 8 (100%) organisations responded.

Three of the eight responding units (38%) reported that they had local guidance that explicitly admits women with GDM for labour care to the MU and about their care (online supplemental table S8). Six out of eight units (75%) reported having guidance about the care of babies born in the MU to a mother with diabetes. Half of the responding units reported that women with diabetes who planned to give birth in the MU would have an individualised care plan written with the woman and a clinician. Six of the eight units (75%) reported that intrapartum care for women with diabetes was different in the MU compared with the OU, most notably because fetal well-being was monitored using intermittent auscultation in the MU rather than continuous electronic fetal monitoring (CEFM). All units reported that women remained in the MU for postnatal care, with only one unit (13%) reporting that the immediate postnatal care of mothers and their babies was different from the OU in terms of blood glucose monitoring. Two units (25%) reported that they were aware of local initiatives to increase access for women with diabetes to midwifery-led care, including a birth choice clinic and a guideline change to admit women with GDM that was well controlled on diet or metformin (online supplemental table S8).

## Discussion

At the time, the study was conducted, national guidance stated that women with GDM or pre-existing diabetes should be advised to plan birth in an OU setting to reduce the risks associated with labour and birth.^3 5^ Our study shows that while admission of women with pre-existing diabetes is rare in MUs, admission of women with GDM to MUs is not uncommon. One-third of units reported admitting at least one woman with diabetes for labour care during the study period, 99% of whom had GDM, with a small number of units reporting significant numbers of women. National guidance has been recently updated, and now states that women who are on medication for diabetes should be advised to plan birth in an OU setting.^6^ In our cohort, 16% of women received medication for their diabetes.

Among women with diabetes, 21% experienced the composite maternal outcome reflecting the increased need for obstetric care, compared with 19% in the comparison group. After adjustment for other factors, this represented a relative increase in risk of 31% for the diabetes group that was not statistically significant, while the absolute risk of experiencing the composite outcome was very similar between the two groups. We found a similar pattern of results for several secondary maternal outcomes, including any transfer during labour or after birth, caesarean birth, perineal trauma, shoulder dystocia and admission for higher level care, with higher absolute risks in the diabetes group, but very small absolute differences, and no statistically significant relative increases in risk. It should be noted that, due to smaller numbers of admissions to MUs during the study period than anticipated, our study was underpowered, even for the primary outcome. However, none of the absolute risks reported appear to indicate any meaningful increased risk of adverse maternal outcome associated with GDM, in this group of women admitted to MUs. Some of the absolute risks of these outcomes are comparable to those found in other studies of women planning birth in MUs.^7 11^

For women with diabetes, at the time of our study, planned birth in an OU setting was in part advised because of proximity to advanced neonatal resuscitation skills. GDM is associated with an increased risk of low 1 min Apgar score.^17^ In our study, we found no difference between the two groups in terms of 5 min Apgar score, with less than 1% of babies in both groups having a score of less than seven. A higher proportion of babies born to women in the diabetes group were admitted to a neonatal unit, although again this difference was not statistically significant. In earlier work about risk factors for neonatal admission among babies born in an MU, the babies of women with maternal pregnancy complications were 1.4 times more likely to be admitted for neonatal care.^18^ In the current study, however, the absolute risk of being admitted to a neonatal unit for babies of women in the diabetes group (3.3%) and (2.4%) in the comparison cohort is also informative. For comparison, the overall incidence of neonatal admission in term babies in the UK in 2016–2017 was around 6%.^19^

Also, of relevance in terms of neonatal well-being are our results on the initiation and continuance of breastfeeding. Previous research has shown that women with GDM, and even more so women who receive insulin, may be less likely to breastfeed.^20^ Breast feeding is associated with short-term and long-term benefits for mothers, including improved lipid and glucose metabolic profiles and reduced incidence of type 2 diabetes .^21 22^ It is also associated with reduced risk of type 2 diabetes, obesity and overweight in the offspring.^22^ In our diabetes cohort, only 8 women (2%) received insulin for their diabetes, but overall 89% initiated breastfeeding and 70% of babies were exclusively breastfed during the newborn protocol for hypoglycaemia prevention. Our data, therefore, compare favourably with national data for Great Britain, which showed that, in 2018–2019, 76% of babies received breastmilk at the first feed, and 72% were still breastfed at discharge.^23^

Our small survey of MUs that reported higher numbers of admissions of women with diabetes, aimed to understand whether these units had specialist guidance in place supporting the admission and care of women with diabetes and their babies, with a view to supporting the dissemination of good practice. A small number of MUs reported having guidelines that explicitly admitted women with diabetes for labour care to the MU or about their care, and half reported the use of individualised care plans for women with diabetes. Most respondents reported having specific guidance about the care of babies born to diabetic mothers in the MU. The main difference reported in the intrapartum care of women with GDM in the MU compared with OUs, was the use of intermittent auscultation to monitor fetal well-being in MUs. National guidance^24^ recommends CEFM in women with pre-existing diabetes and GDM requiring medication but makes no explicit reference to women with GDM not requiring medication. The use of CEFM in labour is associated with an increase in caesarean and instrumental births, with no clear reduction in measures of neonatal well-being such as cerebral palsy or infant mortality.^25^ Intermittent auscultation is part of a package of care in MUs that for women at low risk of complications is of benefit in terms of maternal outcomes, with no adverse impact on neonatal outcomes.^26^ Care in an MU has also been demonstrated to increase women’s satisfaction with care.^27^ Women with GDM have been shown to reflect negatively on their care, particularly in relation to their capacity for personal autonomy and agency.^28^ For those women, care in an MU has the potential to bring a number of benefits.

Finally, with regard to diabetes screening, this study was carried out in a period when services still had not returned to normal following the COVID-19 pandemic. NICE^3^ guidelines recommend an OGTT in women at risk of developing GDM. Women with pre-existing diabetes should be offered an HbA1c test at their first antenatal ‘booking’ appointment and women diagnosed with GDM should be offered an HbA1c test as soon as possible once GDM is confirmed. During the COVID-19 pandemic, guidance for GDM screening was changed to recommend stopping the use of the OGTT in favour of testing at-risk women with HbA1c and fasting or random plasma glucose at booking and 28 weeks.^29^ This change came about to try and minimise the risk of exposure to COVID-19 by accommodating social distancing.^30^ In our study, 14% of women had only an Hba1C test in pregnancy without pre-existing diabetes, suggesting that in those units diabetes screening practice may have changed during the COVID-19 pandemic. Further research may be of value to determine the most effective and acceptable approach to diabetes screening.

The positive outcomes for the women with diabetes and their babies in our study are in all likelihood a result of careful selection by midwives and other clinicians in the context of discussion about planned place of birth and potential admission to an MU, and self-selection by women themselves who have a preference for midwifery-led care. Most women in our diabetes cohort were admitted to AMUs, on the same site as specialist obstetric and neonatal services, with low numbers of FMUs reporting admission of women with diabetes. In the UK, just over 87% of pregnancies in women with diabetes are affected by GDM, 5% by type 2 diabetes and just over 7% by type 1.^3^ The diabetes cohort in this study is clearly not representative of the general UK population of women with diabetes in pregnancy. We are not aware of reliable evidence about the demographic and clinical characteristics of women with GDM in the UK, but it seems likely that our cohort were ‘selected’ for admission to an MU and, therefore, may not be representative of the population of women with GDM. Our results support the recent change in guidelines,^6^ suggesting that for carefully selected women with diet-controlled GDM, and for some on metformin, planned birth in an AMU may have benefits in terms of providing choice for women, with beneficial maternal and neonatal outcomes.

### Strengths and limitations

The main strength of this study is its national population-based design, which reduces the risk of the biases associated with local, hospital-based studies. Most MUs in the UK participated in the study (93% of eligible units), with 90% response to monthly report requests and complete data returned for over 87% of women reported, reducing the possibility of selection bias.

Our aim was to compare outcomes for women admitted to MUs with a diagnosis of diabetes in the current pregnancy with other women admitted to the same units for labour care. Based on this aim and the data we collected, we are not able to compare directly with outcomes for similar women admitted to obstetric-led care.

Women with diabetes in their current pregnancy and admitted for labour care to an MU were identified by UKMidSS reporters in each unit, who also performed data entry. UKMidSS systems involve regular communication with reporters to ensure that eligible women are not missed, and the overall prevalence of diabetes among women admitted to MUs was consistent with estimates from some earlier studies, but it is nevertheless still possible that some women who had a diagnosis of diabetes in the current pregnancy may have been missed. Furthermore, national guidance about diabetes screening changed during the COVID-19 pandemic,^29^ with some evidence from our study that some units may have continued this practice beyond the pandemic. In the light of evidence to suggest that HbA1C testing and plasma fasting glucose may be poor at screening for GDM,^31 32^ we cannot exclude the possibility that a small number of women in our comparison group might have had undiagnosed GDM.

Our diabetes cohort included only three women with pre-existing diabetes and only eight women (12%) who received insulin for diabetes. Most (93%) of the diabetes group were admitted to AMUs and were likely to have been selected for admission in ways that we may not have measured. The generalisability of our results should, therefore, be considered in that light and are most likely to be of relevance to women with diet-controlled GDM or those on metformin.

Finally, when planning this study, we estimated the likely sample size, based on 1-year data collection, using estimates from previous studies about the likely prevalence of diabetes among women admitted to MUs and the likely total number of admissions to MUs. The overall number of admissions to MUs over the study period was significantly lower than expected. The reasons for this are likely to be multifactorial and have not been the subject of robust research, but there is some evidence that staff shortages and redeployment of staff towards hospitals during and after the COVID-19 pandemic may have contributed to this.^33 34^ In addition, the recommendation of induction of labour at 37 and 38+6 weeks respectively in women with uncomplicated type 1 or type 2 diabetes and by 40+6 for women with uncomplicated GDM, might have influenced planned place of birth for women with diabetes.^3^ Largely as a consequence of overall lower admissions, the sample size for our diabetes and comparison cohorts was lower than anticipated, and our study was, therefore, underpowered for the primary outcome. While this is a limitation, and our findings about RRs should be treated with caution, the evidence provided about absolute risks of a range of relevant maternal and neonatal outcomes is of benefit for women and those supporting them in decisions about the planned place of birth.

## Conclusions

With increasing rates of diabetes and GDM worldwide,^1^ the findings of this study are important because they provide evidence that selected women with well-controlled GDM may safely plan birth in an AMU. With clear admission criteria and careful care planning, access to an MU provides an opportunity to increase choice, reduce intervention and improve outcomes for these women.

## supplementary material

10.1136/bmjopen-2024-087161 online supplemental table 1

## Data Availability

Data are available on reasonable request.
