# Supplementary material for: Outcomes for women with diabetes admitted for labour care to midwifery units in the UK: a national prospective cohort study and survey of practice using the UK Midwifery Study System (UKMidSS)
Source: BMJ Open. 2024 Dec 3;14(12):e087161. doi: 10.1136/bmjopen-2024-087161 (PMC11624763; doi:10.1136/bmjopen-2024-087161)
Supplement: online supplemental table 1 [file bmjopen-14-12-s001.docx]

Supplementary tables

***Table S1: Categorisation of covariates***

| **Type of characteristic**  **Variable (unit or how derived)** | **Categorisation** |
| --- | --- |
| **Maternal social demographic** |  |
| Maternal age (years) | Under 20; 20-24;25-29; 30-34; 35-39; ≥40 |
| Ethnic group | White; Asian; Black; Other |
| Socioeconomic status (derived from woman’s occupation, or partner’s if woman not in work or with unrecorded/uncodable occupation, using three class NS-SEC) | Higher managerial, admin, prof; Intermediate; Routine and manual; Unemployed/student; Employment status not recorded |
| Area deprivation quintile (derived from women’s postcode entered by midwives in to postcode look-up website which returned score from Children in Low-income Families Measure, converted into quintiles) | 1st (least deprived); 2^nd^; 3^rd^; 4^th^; 5th (most deprived) |
| Smoking status | Non-smoker during pregnancy; Smoker during pregnancy; Not recorded |
| **Pre-existing clinical factors** |  |
| Previous pregnancies ≥24 weeks’ gestation | 0; 1; 2; ≥3 |
| Body mass index (BMI) at booking (kg/m^2^) | <18·5; 18·5-24·9; 25-29·9; 30-35·0; >35·0; Not recorded |
| Pre-existing medical risk factors (see Table 2) | None; One or more |
| Problems in a previous pregnancy (see Table 2) | None; One or more |
| **Clinical factors arising during pregnancy** |  |
| Current pregnancy problem (see Table 2) | None; One or more |
| Gestation at admission (weeks) | 36-37; 38; 39; 40; 41-42 |
| **Intrapartum-related** |  |
| Immersion in water during labour | No; Yes |
| Transfer during labour or after birth (see Table S5) | No; Yes |
| Augmentation | No; Yes |
| Epidural/spinal | No; Yes |
| General anaesthetic | No; Yes |
| **Birth-related** |  |
| Birthweight (g) | <3000; 3000-3499; 3500-3999; ≥4000 |
| Birth in water | No; Yes |
| Mode of birth | Spontaneous vertex; Vaginal breech; Ventouse; Forceps; Caesarean |
| Active management of third stage | No; Yes |

***Table S2. Diabetes testing in pregnancy***

|  | **Diabetes**  **n=420** | |
| --- | --- | --- |
|  | **n** | **%** |
| **OGTT during pregnancy** |  |  |
| Yes | 315 | 75.9 |
| No | 100 | 24.1 |
| Missing | 5 |  |
| **Number of OGTTs^1^** |  |  |
| One | 277 | 88.2 |
| More than one | 37 | 11.8 |
| Missing | 1 |  |
| **1^st^ OGTT gestation^1^** |  |  |
| <24 weeks | 54 | 17.3 |
| 24-28 weeks | 197 | 63.1 |
| >28 weeks | 61 | 19.6 |
| missing | 3 |  |
| **1^st^ OGTT value^1^** |  |  |
| <=7.7mmol/l | 75 | 27.6 |
| >=7.7 mmol/l | 197 | 72.4 |
| Missing | 4 |  |
| **Latest OGTT gestation^2^** |  |  |
| <=28 weeks | 20 | 54.1 |
| >28 weeks | 17 | 45.9 |
| Missing | 0 |  |
| **Latest OGTT value^2^** |  |  |
| <7.8mmol/l | 12 | 35.3 |
| >=7.8 mmol/l | 22 | 64.7 |
| Missing | 3 |  |
| **HbA1C during pregnancy** |  |  |
| Yes | 214 | 48.7 |
| No | 203 | 51.3 |
| Missing | 3 |  |
| **Number of HbA1C** |  |  |
| One | 111 | 48.1 |
| More than one | 103 | 51.9 |
| Missing | 3 |  |
| **1^st^ HbA1C gestation^3^** |  |  |
| =<13 weeks | 117 | 54.7 |
| >13 weeks | 97 | 45.3 |
| missing | 0 |  |
| **1^st^ HbA1C level^3^** |  |  |
| <=47 | 210 | 98.1 |
| >47 | 4 | 1.9 |
| Missing | 0 |  |
| **Last HbA1C gestation^4^** |  |  |
| =<13 weeks | 31 | 30.1 |
| >13 weeks | 72 | 69.9 |
| missing | 0 |  |
| **Latest HbA1C level** |  |  |
| <=47 | 101 | 98.1 |
| >47 | 2 | 1.9 |
| **Missing** | 0 |  |
| OGTT= oral glucose tolerance test  ^1^ Denominator changed to number of women who had OGTT  ^2^ Denominator changed to number of women who had second OGTT  ^3^ Denominator changed to number of women who Hba1C  ^4^ Denominator changed to number of women who had last HbA1C | | |

***Table S3. Diabetes medication in pregnancy***

|  | **Diabetes**  **n=420** | |
| --- | --- | --- |
|  | **n** | **%** |
| **Medication during pregnancy** |  |  |
| Yes | 65 | 15.6 |
| No | 353 | 84.4 |
| Missing | 2 |  |
| **Medication type^1^** |  |  |
| Metformin | 57 | 87.7 |
| Insulin | 2 | 3.1 |
| Both | 6 | 9.2 |
| ^1^ Denominator change to women who received a medication in pregnancy for diabetes | | |

***Table S4: Maternal blood glucose monitoring in labour***

|  | **Diabetes**  n=420 | |
| --- | --- | --- |
|  | n | % |
| **Documented plan for monitoring in labour** |  |  |
| Yes | 247 | 59.1 |
| No | 171 | 40.9 |
| Missing | 2 |  |
| **Glycaemia monitoring** |  |  |
| Yes | 226 | 45.9 |
| No | 192 | 54.1 |
| Missing | 2 |  |
| **Reason for not monitoring** |  |  |
| Short labour | 128 | 68.7 |
| Declined | 8 | 4.2 |
| Not required | 11 | 5.7 |
| Other* | 4 | 2.1 |
| Not recorded | 37 | 19.3 |
| **Monitoring type** |  |  |
| Self-monitoring | 24 | 10.7 |
| Mixed Staff and self | 58 | 25.8 |
| Staff | 143 | 63.5 |
| Missing | 1 |  |
| *****Other included not available in the MLU (2), transfer to obstetric unit (1) and misdiagnosed (1) | | |

*Table S5: Labour progress, complications and care*

|  | **Diabetes**  **n=420** | | **Comparison**  **n=411** | | **All**  **n=831** | |
| --- | --- | --- | --- | --- | --- | --- |
|  | **n** | **%** | **n** | **%** | **n** | **%** |
| **Immersion in water** |  |  |  |  |  |  |
| Yes | 163 | 38.9 | 147 | 35.9 | 310 | 37.4 |
| Missing | 1 |  | 1 |  | 2 |  |
| **Transfer during labour or after birth^1^** |  |  |  |  |  |  |
| Not transferred | 315 | 75.2 | 317 | 77.1 | 632 | 76.1 |
| Missing | 1 |  | 0 |  | 1 |  |
| **Transfers before birth (total)** | 80 | 19.0 | 77 | 18.7 | 157 | 18.9 |
| Abnormal blood glucose | 2 |  | 0 |  | 2 |  |
| Maternal tachycardia | 0 |  | 2 |  | 2 |  |
| Hypertension | 5 |  | 3 |  | 8 |  |
| Maternal pyrexia | 3 |  | 2 |  | 5 |  |
| Significant meconium | 3 |  | 6 |  | 9 |  |
| 1^st^ stage delay | 17 |  | 8 |  | 25 |  |
| 2nd stage delay | 8 |  | 10 |  | 15 |  |
| Epidural/pain relief | 16 |  | 18 |  | 34 |  |
| 1st stage FHR concerns | 11 |  | 11 |  | 22 |  |
| 2nd stage FHR concerns | 6 |  | 10 |  | 16 |  |
| Other before birth | 9 |  | 7 |  | 16 |  |
| **Transfers after birth (total)** | 24 | 5.7 | 17 | 4.1 | 41 | 4.9 |
| PPH | 7 |  | 4 |  | 11 |  |
| Maternal Tachycardia | 1 |  | 1 |  | 2 |  |
| Hypertension | 2 |  | 0 |  | 1 |  |
| Retained placenta | 2 |  | 5 |  | 7 |  |
| Perineal repair | 10 |  | 5 |  | 15 |  |
| Other after birth | 2 |  | 2 |  | 4 |  |
| Missing | 1 |  | 0 |  | 1 |  |
| **Augmentation** |  |  |  |  |  |  |
| Yes | 25 | 5.7 | 31 | 7.5 | 56 | 6.7 |
| Missing | 1 |  | 0 |  | 1 |  |
| **Epidural/spinal** |  |  |  |  |  |  |
| Yes | 58 | 13.8 | 58 | 14.1 | 116 | 14.0 |
| Missing | 1 |  | 0 |  | 1 |  |
| **General anaesthetic** |  |  |  |  |  |  |
| Yes | 1 | 0.2 | 3 | 0.7 | 4 | 0.48 |
| Missing | 2 |  | 0 |  | 2 |  |
| **Active management of third stage** |  |  |  |  |  |  |
| Yes | 340 | 81.2 | 326 | 79.5 | 666 | 80.3 |
| Missing | 1 |  | 1 |  | 2 |  |
|  | | | | | | |

***Table S6: Secondary maternal outcomes***

|  | Events | Births |  | | Unadjusted | | | Adjusted ^a^ | |
| --- | --- | --- | --- | --- | --- | --- | --- | --- | --- |
|  | n | n | % | 95% CI | RR | 99% CI | RR | | 99% CI |
| **Any transfer (during labour or after birth)** |  |  |  |  |  |  |  | |  |
| Comparison Group | 94 | 411 | 22.9 | 18.9-27.2 | 1 |  | 1 | |  |
| Diabetes Group | 104 | 419 | 24.8 | 20.8-29.2 | 1.09 | 0.70-1.68 | 1.27 | | 0.88-1.82 |
| **Augmentation** |  |  |  |  |  |  |  | |  |
| Comparison Group | 31 | 411 | 7.5 | 5.2-10.5 | 1 |  | 1 | |  |
| Diabetes Group | 25 | 419 | 6.0 | 3.9-8.7 | 0.79 | 0.38-1.66 | 0.98 | | 0.55-1.75 |
| **Instrumental birth** |  |  |  |  |  |  |  | |  |
| Comparison Group | 33 | 411 | 8.0 | 5.6-11.1 | 1 |  | 1 | |  |
| Diabetes Group | 28 | 420 | 6.7 | 4.5-9.5 | 0.83 | 0.42-1.67 | 0.87 | | 0.46-1.65 |
| **Caesarean birth** |  |  |  |  |  |  |  | |  |
| Comparison Group | 20 | 411 | 4.9 | 3.0-7.4 | 1 |  | 1 | |  |
| Diabetes Group | 24 | 420 | 5.7 | 3.7-8.3 | 1.1 | 0.56-2.49 | 1.74 | | 0.55-5.52 |
| **Straightforward vaginal birth^b^** |  |  |  |  |  |  |  | |  |
| Comparison Group | 346 | 411 | 84.2 | 80.3-87.6 | 1 |  | 1 | |  |
| Diabetes | 349 | 420 | 83.1 | 79.2-86.6 | 0.99 | 0.92-1.06 | 0.95 | | 0.88-1.03 |
| **Immersion in water** |  |  |  |  |  |  |  | |  |
| Comparison Group | 147 | 410 | 35.9 | 31.2-40.7 | 1 |  | 1 | |  |
| Diabetes Group | 163 | 419 | 38.9 | 34.2-43.8 | 1.09 | 0.87-1.35 | 1.22 | | 0.94-1.55 |
| **Birth in water** |  |  |  |  |  |  |  | |  |
| Comparison Group | 91 | 357 | 25.5 | 21.0-30.3 | 1 |  | 1 | |  |
| Diabetes Group | 98 | 367 | 26.7 | 22.2-31.5 | 1.05 | 0.81-1.35 | 1.01 | | 0.75-1.37 |
| **Third/fourth degree perineal trauma** |  |  |  |  |  |  |  | |  |
| Comparison Group | 13 | 411 | 3.2 | 1.7-5.3 | 1 |  | 1 | |  |
| Diabetes Group | 18 | 420 | 4.3 | 2.6-6.7 | 1.36 | 0.59-3.13 | 1.54 | | 0.63-3.78 |
| **PPH>=1.5L** |  |  |  |  |  |  |  | |  |
| Comparison Group | 15 | 411 | 3.7 | 2.1-5.9 | 1 |  | 1 | |  |
| Diabetes Group | 10 | 420 | 2.4 | 1.1-4.3 | 0.65 | 0.22-1.96 | 0.57 | | 0.14-2.24 |
| **Shoulder dystocia** |  |  |  |  |  |  |  | |  |
| Comparison Group | 5 | 410 | 1.2 | 0.4-2.8 | 1 |  | 1 | |  |
| Diabetes Group | 5 | 419 | 1.2 | 0.4-2.8 | 0.98 | 0.18-5.47 | 1.67 | | 0.95-29.5 |
| **Maternal blood transfusion** |  |  |  |  |  |  |  | |  |
| Comparison Group | 6 | 411 | 1.5 | 0.5-3.1 | 1 |  | 1 | |  |
| Diabetes Group | 2 | 420 | 0.5 | 0.1-1.7 | 0.33 | 0.48-2.23 | 0.37 | | 0.04-3.56 |
| **Maternal admission for higher level care** |  |  |  |  |  |  |  | |  |
| Comparison Group | 15 | 411 | 3.7 | 2.1-5.9 | 1 |  | 1 | |  |
| Diabetes Group | 20 | 419 | 4.8 | 2.9-7.2 | 1.31 | 0.71-2.42 | 1.23 | | 0.49-3.10 |
| ^a^ Adjusted for: parity, maternal age, ethnicity, socioeconomic status, area deprivation quintile, smoking status, birthweight, gestation at admission, BMI, previous pregnancy complications, medical risk factors where appropriate. For some outcomes, not adjusted for all potential confounders because of small numbers.  ^b^ Defined as birth without forceps, ventouse or caesarean, with no third/fourth degree perineal tear and no blood transfusion | | | | | | | | | |

***Table S7: Neonatal hypoglycaemia monitoring and feeding method***

|  | **Diabetes**  **n=420** | | **Comparison**  **N=411** | |
| --- | --- | --- | --- | --- |
|  | **n** | **%** | **n** | **%** |
| **Hypoglycaemia monitoring** |  |  |  |  |
| Yes | 392 | 93.8 | 15* | 3.6 |
| No | 26 | 6.2 | 395 | 96.3 |
| Missing | 2 |  | 1 |  |
| **Exclusive breastfeeding during blood sugar protocol** |  |  |  |  |
| Yes | 279 | 71.4 | 11 | 73.3 |
| No | 112 | 28.6 | 4 | 26.7 |
| Missing | 1 |  | 0 |  |
| *Reason for blood sugar monitoring: Pre-term (2), lethargy (1), SGA (6), Low birth weight (2), perinatal acidosis (1), suspected/confirmed sepsis (3) | | | | |

***Table S8: Diabetes study survey***

|  | **Units**  **n=8** | |
| --- | --- | --- |
|  | **n** | **%** |
| **Do you have guidance that explicitly admits women with diabetes (either gestational or pre-existing) for labour care to the midwifery unit?** | | |
| No | 5 | 62.5 |
| Yes | 3 | 37.5 |
| **Do you have any specific guidance about the care of women with diabetes (either gestational or pre-existing) in the midwifery unit?** | | |
| No | 5 | 62.5 |
| Yes | 3 | 37.5 |
| **Do you have any guidance about the care of babies born in a midwifery unit to a mother with diabetes?** | | |
| No | 2 | 25 |
| Yes | 6 | 75 |
| **Do women with diabetes who labour in a MLU have an individualised birth plan written with the woman and another clinician (eg obstetrician and/or consultant midwife and/or other senior midwife)?** | | |
| No | 4 | 50 |
| Yes | 4 | 50 |
| **Does the intrapartum care for women with diabetes in the midwifery unit differ from care for similar women in the obstetric unit?** | | |
| No | 2 | 25 |
| Yes^1^ | 6 | 75 |
| **Do women with diabetes who give birth in the midwifery unit stay there for postnatal care?** | |  |
| No | 0 | 0 |
| Yes | 8 | 100 |
| **Does the immediate postnatal care for women with diabetes who give birth in the midwifery unit differ from care for similar women in the obstetric unit?** | | |
| No | 7 | 87.5 |
| Yes^2^ | 1 | 12.5 |
| **Does care for babies of mothers with diabetes in the midwifery unit differ from the care for babies of similar mothers in the obstetric unit?** | | |
| No | 7 | 87.5 |
| Yes^2^ | 1 | 12.5 |
| **Are you aware of any local initiatives to increase access for women with diabetes to midwifery-led care?** | | |
| No | 6 | 75 |
| Yes^3^ | 2 | 25 |
| ^1^ Women in the MLU have IA instead of CEFM (5), hourly blood glucose in labour(1)  ^2^ Blood glucose monitoring  ^3^Birth choice clinic and guidelines change for women with diet/metformin GDM who are well controlled. | | |
