# Supplementary material for: Outcomes for women with diabetes admitted for labour care to midwifery units in the UK: a national prospective cohort study and survey of practice using the UK Midwifery Study System (UKMidSS)
Source: BMJ Open. 2024 Dec 3;14(12):e087161. doi: 10.1136/bmjopen-2024-087161 (PMC11624763; doi:10.1136/bmjopen-2024-087161)
Supplement: Supplementary file 2 [file bmjopen-2024-087161.reviewer_comments.pdf]

PEER REVIEW HISTORY

BMJ Open publishes all reviews undertaken for accepted manuscripts. Reviewers are asked to complete a checklist review form and are provided with free text boxes to elaborate on their assessment. These free text comments are reproduced below.

ARTICLE DETAILS

Title (Provisional)

Outcomes for women with diabetes admitted for labour care to midwifery units in the UK: A national prospective cohort study and a survey of practice using the UK Midwifery Study System (UKMidSS)

Authors

Morelli, Alessandra; Smith, Lisa; Karia, Amar; Marshall, Amber; Plachcinski, Rachel; Tyler, Wendy; Rowe, Rachel

---

VERSION 1 - REVIEW

**Reviewer**	**1**
**Name**	**Higueras, Maria Velo**
**Affiliation**	**Robert Gordon University**
**Date**	**18-Jun-2024**
**COI**	**None to declare**

Thank you for the opportunity to review this paper. I would like to congratulate the authors for a very timely, relevant and well conducted study that will surely influence current clinical practice in relation to birthplace options for GDM.

A couple of minor comments:

- in line 50-53, authors state "The diabetes cohort in this study is clearly not

representative of the general UK population of women with diabetes in pregnancy, nor even of the population of women with GDM". Further explanation here would enhance this point for the reader who is not familiar with the demographic characteristics of the UK population of women with GDM.

- in line 23-24, authors stated "74% of all women reported with a diagnosis of diabetes in AMUs were 'clustered' in 12 units." Out of curiosity, was there any geographical pattern for this cluster? (e.g. AMU located in close proximity to each other and potentially influencing neighbouring practices?

**Reviewer**	**2**

**Name**	**Hatunic, Mensud**
**Affiliation**	**Mater Misericordiae Hospitall, Dublin**
**Date**	**08-Aug-2024**
**COI**	**None**

This paper has high clinical importance the tables and figure are well presented and important. The paper is well written and easy to read.

I have some suggestions:

1. Abstract page 3: sentence line 8-10 needs to be revised

2. Do you have data how long was breastfeeding?

2. Page 21- Figure dose not project well

4. Add OGTT values for diagnosis of GDM

5. Why significant number of women had repeated OGTT

6. You had 17% pf participants OGTT <24 weeks, this needs comments

7. Page 27, Table S7 is important and should be in the main part of the paper

VERSION 1 - AUTHOR RESPONSE

Reviewer: 1

Dr. Maria Velo Higueras, Robert Gordon University

Comments to the Author:

Thank you for the opportunity to review this paper. I would like to congratulate the authors for a very timely, relevant and well conducted study that will surely influence current clinical practice in relation to birthplace options for GDM.

Response: Thank you for reviewing our paper. We appreciate your comments.

A couple of minor comments:

- in line 50-53, authors state "The diabetes cohort in this study is clearly not

representative of the general UK population of women with diabetes in pregnancy, nor even of the population of women with GDM". Further explanation here would enhance this point for the reader who is not familiar with the demographic characteristics of the UK population of women with GDM.

Response: Thank you for this comment. There is little recent evidence about the demographic characteristics of the UK population of women with GDM. However, we added a sentence explaining the proportion of women with diabetes in the UK that have GDM, type 1 and type 2 diabetes.

- in line 23-24, authors stated "74% of all women reported with a diagnosis of diabetes in AMUs were ‘clustered’ in 12 units." Out of curiosity, was there any geographical pattern for this cluster? (e.g. AMU located in close proximity to each other and potentially influencing neighbouring practices?

Response: There was some evidence that some units with higher numbers of women with a diagnosis of diabetes were in the same NHS region. We have added this to the paper.

Reviewer: 2

Dr. Mensud Hatunic, Mater Misericordiae Hospitall, Dublin

Comments to the Author:

This paper has high clinical importance the tables and figure are well presented and important. The paper is well written and easy to read.

Response: Thank you for reviewing our paper. We appreciate your comments.

I have some suggestions:

1. Abstract page 3: sentence line 8-10 needs to be revised

Response: Thank you. We have revised the abstract also taking into account the editorial recommendations.

2. Do you have data how long was breastfeeding?

Response: Unfortunately, we did not collect data about breastfeeding duration.

2. Page 21- Figure dose not project well

Response: Apologies for this. We submitted the figure in the format requested by the journal. We will make sure that this is addressed at the editing stage. The figure otherwise is visible on page 8 of the manuscript.

4. Add OGTT values for diagnosis of GDM

Response: Thank you for your comment. We presented OGTT values as a dichotomous variable using the cut- off based on the diagnostic criteria from the NICE (NICE, 2015) guidelines, but chose not to represent the range of OGTT values for diagnosis of GDM as there is already a lot of information in the paper and it is not clear what added value this would bring.

5. Why significant number of women had repeated OGTT

Response: We did not specifically ask about the indication for a repeated OGTT in our study. However, women who had gestational diabetes before and have a high risk of developing it, should be offered an OGTT as soon as possible after booking and a further OGTT at 24-28w, if the result of the first OGTT is normal (NICE, 2015)

6. You had 17% pf participants OGTT <24 weeks, this needs comments

Response: See comment above

7. Page 27, Table S7 is important and should be in the main part of the paper

Response: Thank you for your comment. Whist we appreciate the importance of table S7, our preference would be to retain this table as a supplementary file, as it doesn’t present results relating to the main objective of the study. We are happy to take editorial guidance as to whether it should be moved to the main body of the paper
